# Ethical issues at the interface of clinical care and research practice in pediatric oncology: a narrative review of parents' and physicians' experiences

**DOI:** 10.1186/1472-6939-12-18

**Published:** 2011-09-27

**Authors:** Martine C de Vries, Mirjam Houtlosser, Jan M Wit, Dirk P Engberts, Dorine Bresters, Gertjan JL Kaspers, Evert van Leeuwen

**Affiliations:** 1 Department of Pediatrics, Leiden University Medical Center, J6-S, PO Box 9600, 2300 RC Leiden, The Netherlands; 2 Department of Medical Ethics and Health Law, Leiden University Medical Center, J1-P, PO Box 9600, 2300 RC Leiden, The Netherlands; 3 Division of Pediatric Oncology/Hematology, VU University Medical Center, Po Box 7057, 1007 MB Amsterdam, The Netherlands; 4 IQ Healthcare, Section Ethics, Philosophy and History of Medicine, UMC St Radboud, PO Box 9101, 114 6500 HB Nijmegen, The Netherlands

## Abstract

**Background:**

Pediatric oncology has a strong research culture. Most pediatric oncologists are investigators, involved in clinical care as well as research. As a result, a remarkable proportion of children with cancer enrolls in a trial during treatment. This paper discusses the ethical consequences of the unprecedented integration of research and care in pediatric oncology from the perspective of parents and physicians.

**Methodology:**

An empirical ethical approach, combining (1) a narrative review of (primarily) qualitative studies on parents' and physicians' experiences of the pediatric oncology research practice, and (2) comparison of these experiences with existing theoretical ethical concepts about (pediatric) research. The use of empirical evidence enriches these concepts by taking into account the peculiarities that ethical challenges pose in practice.

**Results:**

Analysis of the 22 studies reviewed revealed that the integration of research and care has consequences for the informed consent process, the promotion of the child's best interests, and the role of the physician (doctor vs. scientist). True consent to research is difficult to achieve due to the complexity of research protocols, emotional stress and parents' dependency on their child's physician. Parents' role is to promote their child's best interests, also when they are asked to consider enrolling their child in a trial. Parents are almost never in equipoise on trial participation, which leaves them with the agonizing situation of wanting to do what is best for their child, while being fearful of making the wrong decision. Furthermore, a therapeutic misconception endangers correct assessment of participation, making parents inaccurately attribute therapeutic intent to research procedures. Physicians prefer the perspective of a therapist over a researcher. Consequently they may truly believe that in the research setting they promote the child's best interests, which maintains the existence of a therapeutic misconception between them and parents.

**Conclusion:**

Due to the integration of research and care, their different ethical perspectives become intertwined in the daily practice of pediatric oncology. Increasing awareness of what this means for the communication between parents and physicians is essential. Future research should focus on efforts that overcome the problems that the synchronicity of research and care evokes.

## Background

Children treated for cancer are increasingly likely to survive. For all childhood cancers combined, 5-year overall survival has improved over the past 30 years from less than 20% to about 75%, due to improved treatment and supportive care [1,2]. A major factor contributing to these advances is the systematic research effort in pediatric oncology. Pediatric oncology has a strong research culture. This is instigated by two circumstances: evidence from research with adults with cancer cannot be generalized to children and childhood cancer is a rarity. If long delays in making evidence-based treatments available to children with cancer are to be avoided, it is important that trials in pediatric oncology recruit a much greater proportion of the patient population than adult cancer trials. As a consequence, most pediatric oncologists are investigators involved in both clinical care and research.

In the setting of pediatric oncology most treatments are given according to national or international protocols which describe in detail the treatment plan for each type of cancer. Protocols represent the best available treatment at a given moment according to the published medical literature, but may also include research components which contain potential improvements of the treatment. Table 1 shows different types of research which are often performed during treatment. As a result of these research efforts, a remarkable proportion of children with cancer - up to 70% of children in the developed world - enrolls in a study during their cancer treatment, as compared to only 1-4% of adult patients [3-5]. Due to the integration of research and care the pediatric oncology practice always faces ethical challenges inherent to research participation.

**Table 1:** Description of different types of research in pediatric oncology

Type of research	Description
Randomized Controlled Trials (RCT)	The random allocation of different treatments to patient-subjects. The best available treatment is compared to one or more regimens that are expected to either improve overall survival or lessen toxicity with equivalence of outcome. Most 'front-line' pediatric cancer studies are phase III randomized controlled trials.

Clinical Controlled Trial (CCT)	Evaluation of singe-arm treatment protocols by clinical and epidemiological data collection and systematic analysis of disease characteristics, actual treatment, treatment results and side-effects. Current treatment results are compared to historical results and to results obtained by other research groups.

Laboratory research using tissue from patients	Unraveling the pathogenesis of childhood cancers, characterization of tumor biology, detection of new treatment targets and identification of novel prognostic factors. For this purpose left over blood, bone marrow or cerebrospinal fluid is used, or additional biological specimens are taken at defined moments during treatment. In fact, permission is often asked for storage of biological materials in a cell bank for future, as yet not specified research.

The aim of this paper is to describe the ethical consequences of the fading boundary between research and care from the perspective of parents and physicians. We present an empirical ethical approach using a narrative review of existing empirical research evidence on parents' and physicians' experiences of the integration of research and care. The use of empirical evidence enhances moral thinking by taking into account the peculiarities and difficulties that ethical challenges pose in practice. The results therefore remain much closer to the particular reality of the ethical consequences than a theoretical paper would permit [6].

## Methodology

### A narrative review within empirical ethics

In ethics, the use of empirical evidence has become more and more popular, leading to a distinct form of applied ethics, namely empirical ethics. Especially in bioethics, this 'empirical turn' is visible [7]. Empirical ethics is a broad category, grasping different interpretations of integrating ethics and empirical research. There is, however, one basic assumption in all sorts of empirical ethics: the study of people's actual moral beliefs, intuitions, behavior and reasoning in a practice yields information that is meaningful for ethics [8]. It denies the structural incompatibility of empirical and normative approaches, and believes in their fundamental complementarity. It is an answer to the critique of bioethics for being too abstract, too general, too dogmatic, as well as too far removed from clinical reality, insensitive to the peculiarities of specific situations.

To gain empirical information, we conducted a narrative review of (primarily) qualitative studies on experiences of parents and physicians in the pediatric oncology research practice. We subsequently confronted these experiences with existing theoretical ethical concepts about (pediatric) research, namely goals of research, informed consent, best interests, equipoise and therapeutic misconception. In other words, the theoretical ethical concepts were the starting point and were subsequently enriched by the emergent themes from the narrative review. The experiences of parents and physicians give us unique insights in the research practice and the way ethical concepts function in this practice. Because we use empirical findings we come much closer to the reality of the ethical challenges faced than a theoretical paper could [6,9].

Two of the authors reported previously on the importance and the methodology of using empirical findings to inform moral thinking [6]. The authors used the same methodology in a paper on discussing infertility risks and semen cryopreservation with male adolescents diagnosed with cancer [10] and in a paper on informed consent in pediatric oncology research [11].

### Literature search

An initial work-up established that the literature was too heterogeneous to permit a systematic review of qualitative studies along the lines proposed by Dixon-Woods [12]. Furthermore, a systematic review would not permit a wide and comprehensive scope and the opportunity to cover a wide range of issues (concepts) within the topic of pediatric oncology research ethics [13]. For these reasons, a narrative review was undertaken.

Studies included in the review were identified by keyword searches of Web of Science, Picarta, Pubmed, Cochrane and EMBASE. Keywords searched included 'oncology', 'clinical trials', 'pediatric*', 'decision making', 'informed consent', 'parents', randomized controlled trials' in combination with 'qualitative study', 'semi-structured', 'ethnograph*' and 'experiences'. Manual searches of other relevant journals (JCO, Pediatric Blood and Cancer, Journal of Pediatric Hematology and Oncology, Pediatrics) and reference lists of primary articles found from initial searches were also conducted.

The focus of the review was on the research types described in Table 1. Other types of research, especially phase I and II studies, are also performed in the pediatric oncology setting. However, the described three types of research have typically become integrated into pediatric oncology practice in such a way that, in contrast with phase I and II studies, almost all patients and their parents are confronted with them at the start of initial treatment. The ethical challenges of phase I and II studies fall beyond the scope of this paper, because these studies are usually applied in second line treatment and are not part of initial treatment protocols.

The median age of children diagnosed with cancer is below 6. Therefore it is assumed in this paper that the child is not competent, and that parental authority and the physician's care are the main factors in determining the best interest of the child. We discussed the difficulties of obtaining assent and consent for research from older children elsewhere [11]. Studies focusing on children's experiences were excluded. In common with other narrative reviews, evaluations of methodological quality were not used to exclude papers.

The search revealed 20 qualitative studies, 1 quantitative study, and 1 combination of a quantitative questionnaire and qualitative interviews. Not all studies focus (only) on the pediatric oncology research context, but (also) on adult and clinical context. These studies were still included because they provided important information on especially physicians' attitudes towards research and their conflicting professional roles of physician and investigator. For this topic, it was not necessary to have a pediatric setting. Table 2 summarizes the 22 study characteristics, including setting (pediatric vs adult, research vs clinical context), methodology applied (interviews, observations, questionnaire) and perspective (parents, physician).

**Table 2:** Summary of study characteristics

Author & Date [reference]	Setting	Methodology	Sample characteristics
Vries e.a. 2010 [11]	Pediatric oncology clinical research	- Retrospective interviews	Physicians n = 15

Kodish e.a. 2004 [23]	Pediatric oncology clinical research	- Observations - Retrospective interviews	Parents n = 137

Wiley e.a. 1999 [24]	Pediatric oncology clinical research	- Questionnaire (retrospective)	Parents n = 192

Stevens e.a. 2002 [25]	Pediatric oncology clinical research	- Retrospective interviews	Parents n = 12

Chappuy e.a. 2010 [27]	Pediatric oncology clinical research	- Retrospective interviews	Parents n = 43

Dermatis e.a. 1990 [28]	Pediatric oncology clinical research	- Questionnaire (retrospective)	Parents n = 61

Levi e.a. 2000 [29]	Pediatric oncology clinical research	- Focus group interviews (retrospective)	Parents n = 22

Van Stuijvenberg e.a. 1998 [31]	Pediatric general practice clinical research	- Questionnaire (retrospective)	Parents n = 181

Tait e.a. 1998 [32]	Pediatric anesthesia clinical research	- Questionnaire (retrospective)	Parents n = 246

Singhal e.a. 2002 [34]	Neonatology clinical research	- Questionnaire (retrospective)	Parents n = 231

Reynolds e.a. 2007 [35]	Pediatric endocrinology clinical research	- Interviews (hypothetical decisions about research)	Parents n = 31

Kupst e.a. 2003 [36]	Pediatric oncology clinical research	- Retrospective interviews	Parents n = 20

Snowdon e.a. 1997 [37]	Neonatology clinical research	- Questionnaire (retrospective) - Retrospective interviews	Parents n = 71

Eiser e.a. 2005 [38]	Pediatric oncology clinical research	- Retrospective interviews	Parents n = 50

Heneghan e.a. 2004 [40]	Pediatric general practice No research	- Focus group interviews	Parents n = 44

McKenna e.a. 2010 [45]	Pediatric oncology clinical research	- Questionnaire (retrospective) - Retrospective interviews	Parents n = 66

Appelbaum e.a. 1982 [46]	Adult psychiatry clinical research	- Observations - Retrospective interviews	Adult patients n = 31

Joffe e.a. 2001 [48]	Adult oncology clinical research	- Questionnaire (retrospective)	Adult patients n = 207

Taylor 1992 [50]	Adult oncology clinical research	- Questionnaire (retrospective) - Retrospective interviews	Physicians n = 101

Taylor e.a. 1994 [51]	Adult & pediatric oncology clinical research	- Questionnaire (retrospective - quantitative study) - Retrospective interviews (n = 43)	Physicians n = 1485

Joffe e.a. 2002 [52]	Adult & pediatric oncology clinical research	- Questionnaire (retrospective) (quantitative study)	Physicians n = 547

Instone e.a. 2008 [61]	Adult gastroenterology clinical research	- Observations - Retrospective interviews	Physicians + nurses, n = 19

The existing (theoretical) ethical concepts about research were studied using ETHX on the web, Philosopher's Index and Bioethics Line. We focused on 'research goals', 'informed consent', 'best interests', 'equipoise' and 'therapeutic misconception'.

Articles and books were included up to January 2011.

## Results and Discussion

Analysis of the 22 studies reviewed revealed four main themes: intertwinement of research and treatment goals, problems with informed consent, promoting best interests in a research setting and therapeutic misconceptions.

### Goals of research and care: who's best interest?

One of the consequences of the fading boundary between research and clinical care is that the goals of research and treatment become intertwined. From an ethical point of view, the goals of treatment and research are fundamentally different [14,15]. In the treatment relationship, the best interests of the individual child prevail when treatment options are discussed. Generation of new knowledge is incidental compared to the overriding goal of providing optimal therapy. In the context of research, the researcher seeks to advance knowledge about what could be the best care of children, as well as to serve other interests like academic merit. Therapeutic benefits to the individual child are, in the research perspective, secondary to the overriding goal of obtaining robust data and new knowledge. Children therefore may have to undergo procedures that are not determined by the goals of treatment, like additional blood samples, spinal taps and (PET-) scans.

The different ethical perspectives of treatment and research can also be illustrated by the different types of ethical principles governing the two activities [14,16,17]. In the context of pediatric treatment the principles of beneficence and non-maleficence dictate the practice, translated in the statement that treatment should be in the best interest of the individual child. Usually parents and physicians share the same ideas on what constitutes the child's best interest, making it possible to use the concept of implicit consent. In the context of research, however, beneficence also involves benefits to others. The prevailing principle therefore is respect for autonomy. Respect for autonomy incorporates two ethical convictions: firstly, that individuals should be treated as autonomous subjects, and secondly, that persons with diminished autonomy are entitled to protection [14] Respect for autonomy demands that subjects voluntarily participate in research, with adequate information and only after explicit consent. In the case of incompetent subjects, like young children, there should be vigorous protection against abuse, and substitute permission should be sought. Due to the integration of research and care, their different ethical perspectives must simultaneously be applied in the daily practice of pediatric oncology. This has consequences for the informed consent process, the promotion of the best interest of the child by parents, and the role of the physician (doctor vs. scientist).

### Informed consent in the pediatric oncology research setting

Joffe and all have shown that the context of pediatric oncology contains many obstacles to a good informed consent process [18], especially when discussions regarding diagnosis and treatment include dialogue about participation in research. Parents have the difficult task to differentiate between research and clinical issues, for example when talking about goals and risks. Informed participation in decision making requires adequate understanding of treatment options, but simultaneously the understanding of the distinction between research and therapeutic intent and of difficult research-related concepts, such as randomization, voluntariness and the risk-benefit ratio. The consent forms involved, explaining concepts and methods, are complex by nature and difficult to understand [19-22]. Studies show that parents frequently have an incomplete understanding of the necessary elements of informed consent in research, especially of the risks, the procedures, the possibility of alternative treatments, the duration of participation, their right to withdraw, and the voluntariness of participation [23-25]. A reason for this incomplete understanding might be that the standard treatment protocols often include research interventions, like extra blood draws, or a randomization, as if these research elements were an integral part of the treatment. For example, the Dutch protocol for acute lymphoblastic leukemia (ALL) states in the parent information form:

*"Almost all children with ALL are treated according to a national protocol of the Dutch Childhood Oncology Group or according to an international protocol. A protocol contains guidelines for research and the mode of treatment" *[26].

This can make parents think that when they consent to the protocol, they consent to the treatment as well as to the research elements, and that it is not an option only to accept the treatment and to decline research participation [27].

The literature also shows that physicians find it difficult to obtain truly informed consent for complex treatment and research proposals from parents who are emotionally distressed because their child has a life threatening condition [18,28]. The extraordinary psychological strain influences physician-parent communication and limits its potential effectiveness, especially when decisions about research participation need to be made within hours or days [29]. The emotional setting of (pediatric) oncology puts in this way great trust on the acting physician who raises treatment and research options [30,31]. In fact, consent for research is often based on the relationship of trust which exists in the care setting between the physician and the parents [28,32].

Consequently, in the pediatric oncology setting true consent from parents for research is difficult to obtain.

### Promoting best interests from a parental point of view

The primary responsibility of parents is to care for and protect their child, also when parents are asked to consider enrolling their child in a pediatric clinical trial [33]. This responsibility makes it difficult for them to think of a research setting as detached from the best interests of their child. Of course parents often state that they are motivated to support research that may improve the chances of future patients. They acknowledge that the participation of other children in former trials has improved treatment to the benefit of their own child and they are prepared to do likewise. But protecting their child is fundamental to the parental role and this shapes how they think about trials [32]. Some form of altruism can play a role in deciding to let a child participate in research, but only with the firm conviction that the research will not pose any harm to the child, and even more, with the prospect that the research will benefit the child [34,35].

Parents will take many different factors into account when deciding whether or not to let their child enter an RCT and will not simply accept randomization because an ethics committee has deemed it permissible. For some parents, randomized trials may represent the prospect of receiving a new treatment with a potentially important direct benefit to their child [36]. If this new treatment is only available within a trial, parents may consent to their child's entry for the chance of receiving the assumed benefits [37]. It can cause them to anticipate regret for not at least trying to get this new treatment through research participation, all from their perspective as guardian of the interests of their child.

The question whether a potential benefit will become available through participation in research is particularly germane. It can be hard for parents to understand that a study involves a new, and in their view potential better therapy for their child, but that research methodology involves randomization, and that the child can also draw the standard therapy [38]. On finding that their child has been allocated to the standard arm, parents sometimes report a sense of missed opportunity, as if the child has been deprived of a known beneficial treatment [32]. This may lead to unwanted tensions between parents and physicians at the outset of a long treatment relationship.

On the other hand, parents can also hesitate to participate because of fears for an 'experimental' arm, of being used as a 'guinea pig', or of a computer choosing what therapy will be given [36].

The treating physician and the investigator are often the same person. This can make parents fear that refusal will have consequences for their future relationship with the physician. Parents frequently indicate that they find it difficult to oppose the proposal of the physician, because they are afraid that this might jeopardize the relationship with the physician, even though consent forms explicitly state the opposite [39,40].

Whatever arm of a trial parents think is better for their child, their preference shows that the idea of clinical equipoise held by the expert medical community is not directly transferable to the parent setting. For parents the different arms of a trial are often not in equipoise. Firstly, because they can hold the conviction that one arm is medically superior [36-38]. Secondly because the arms may differ in duration or in the amount of extra visits or blood draws. The personal context of a family then determines whether or not a trial arm fits to this family. For example, parents may prefer one trial arm because they live long distance from the hospital and the preferred arm contains less extra visits. *Parental *equipoise would be the point at which the parents are 'maximally uncertain' regarding the relative efficacy, safety and fittingness to their personal situation of comparator interventions. Parental equipoise has not been described before, but could be seen as the 'proxy version' of patient equipoise. When Freedman defined (clinical) equipoise, he stated that it is the expert medical community that ought to be in equipoise [41]. In the literature however, it is argued that not only the medical expert community should be in equipoise, but also the trial participants themselves [42,43]. London argues that this has consequences for the informed consent process:

*"There may be reasons that might lead a potential trial participant to prefer one treatment over another even though expert opinion is conflicted. (...) When this is the case, participating in a clinical trial may not be a permissible option for that patient. (...) This example (...) provides a clear focus for the goals of the informed consent process: to ensure that only those individuals participate in research who see the clinical trial as a reasonable option in light of the conflict or uncertainty that exists in expert medical opinion." *[[42], p.584].

This resembles Hans Jonas' argument that the people who should be enrolled in a clinical trial should be the ones who most identify with the cause of research [44]. The mentioned literature [32-38] shows that parental equipoise is often very difficult to reach, which leaves parents with the agonizing situation of wanting to do what is best for their child, while not knowing whether research participation is the best course of action to achieve this and being fearful of making the wrong decision [45].

### Therapeutic Misconception

Parents can mix up research and treatment goals [23-25,27]. Failure to appreciate the difference between the context of research and treatment, and therefore inaccurately attributing therapeutic intent to research procedures is called 'the therapeutic misconception' [46,47]. It refers to the research subject's failure to appreciate that the aim of research is to obtain scientific knowledge, and that any benefit to the subject is a side product of that goal. Though the data are scarce, there is evidence that many trial participants in oncology hold therapeutic misconceptions [23,48]. One study found that 40-80% of subjects show basic misunderstandings of the research trial design [49].

Due to the fading boundary between research and care in pediatric oncology therapeutic misconceptions can easily arise. This puts another burden on the process of informed consent during which parents will have to be made aware that the proposed treatment is not selected only because of the individual needs of the patient.

### The physician's perspective: doctor vs. scientist

Not only parents find it difficult to distinguish treatment and research goals. Physicians and other health personnel may also experience problems when research and clinical care are performed simultaneously. The traditional division of tasks in clinical or research-related is challenged by the emergence of randomized controlled trials (RCTs) and clinical controlled trials (CCTs, see also Table 1) [50]. Physicians may experience tension between their roles as clinicians and scientists, since the latter defies the traditional definition of their core task to place the best interests of patients first. As a solution most oncologists, even those with substantial trial involvement, focus first of all on the possible benefit to their immediate patient and not on the theoretical benefit of future patients. In this way they adopt the perspective of a therapist rather than that of a researcher [51]. Studies show that many oncologists really feel that research participation is in the best interest of the individual child [11,52]. This can be understood in two ways. Firstly, physicians feel confident that trials are not *harmful*, and that control by an Institutional Review Board (IRB) protects the child. Secondly, physicians believe that being in a trial, independently of the arm the patient is in, is even better than receiving the same treatment outside of the trial [11]. Enrolling children in clinical trials would ensure that they receive the best treatment. The website of the M.D. Anderson Cancer Center, one of the leading centers for cancer research and care, states underneath a list of diseases for which clinical trials are available:

*"When you are offered to participate in a clinical trial, your doctor has decided that the best treatment for your condition is provided in that trial" *[53].

This suggests that trials are viewed not only as a way to improve treatment in the future, but also as the best treatment for current patients. Pediatric oncologists tend to view trial protocols as clinical practice guidelines [52]. The statements by many leaders in oncology that clinical trials represent the optimum care for cancer patients are also an expression of this view [54,55].

It could be argued that a RCT does preserve the basic duty to act in the best interest of the child because of clinical equipoise. After all, no subject is randomized to a treatment known to be inferior to the standard treatment. But the fundamental difference between research and treatment is that the treatment setting requires an experienced clinician who selects and monitors the treatment taking into account individual, person-specific factors. The setting of a RCT requires that once a child is thought eligible for participation, the investigator renounces patient specific considerations and uses randomization and a meticulously followed protocol in order to get generalizable results. (Of course taking into account that a child will be removed from a study if it is not in the its interests to remain in the study and also that treatments will be adjusted if a child is too unwell to tolerate them.) Especially when experimental treatments are evaluated, the risks and benefits in randomized trials are less fixed than those in standard medical care. It is the hallmark of randomized studies that it is never known in advance what the actual risks and benefits will be: only after the completion of a study one genuinely knows which arm of the trial showed the best results and whether or not participants were exposed to extra risks and burdens in the intervention arm. Kumar et al. [56] described 126 RCTs within the setting of the Children's Oncology Group. They showed that new (experimental) treatments are as likely to be inferior as they are to be superior to standard treatments. Most pediatric oncology trials have Data and Safety Monitoring Plans (DSMP) and suspension rules for the very purpose of dealing with unexpected risks and outcomes.

It has been argued that trial participation is beneficial as compared to non-participation because of the strict adherence to well-defined protocols. Various authors have shown that the use of a treatment protocol improves the end result of that treatment [57,58]. This would be due to the explicit description of treatment phases and follow up and to strict guidelines indicating how to deal with side effects and relapses. For CCTs one could conclude that participation is beneficial, since CCTs are single arm studies in which the best available treatment is laid down in a protocol (to be able to compare treatment results to historical results). But it is more difficult to apply this to RCTs. A Cochrane review assessed whether there were beneficial effects from participating in RCTs [59]. The outcomes of patients who participated in RCTs were compared with outcomes of patients who received similar clinical interventions outside the RCT. On average, the outcomes were similar, suggesting that participation in RCTs does not result in improved outcomes. Peppercorn et al. therefore state:

"*Despite widespread belief that enrollment in clinical trials leads to improved outcomes in patients with cancer, there are insufficient data to conclude that such a trial effect exists. Until such data are available, patients with cancer should be encouraged to enroll in clinical trials on the basis of trials' unquestioned role in improving treatment for future patients" *[60].

The belief that enrolling children in RCTs ensures that they receive state-of-the-art treatment and that participation is best for the individual child is therefore an example that the therapeutic misperception may also be fostered by physicians [61].

In conclusion, the physician-investigator has a 'hybrid' identity. He serves two different goals: the best interest of the patient and scientific progress (and thereby the best interest of future patients). Physicians are more likely to prefer the perspective of a therapist over that of a researcher, and consequently they may truly come to believe that in the research setting they promote the child's best interests. With this position physicians potentially promote the existence of a therapeutic misconception between them and parents.

Some authors contend that, to reduce misunderstandings about the nature and purpose of research, physician-investigators should restrict themselves to being scientists only and not doctors [62]. In this way the ethical insistence on a clear boundary between research and clinical activity can be secured. This could also partially be achieved by letting research nurses carry out informed consent conversations with parents and children. In both situations, the role of the researcher and the goal of research may be clearer. This solution would however require a fundamental change in the pediatric oncology practice and as such might raise its own problems, for instance regarding the available health personnel and communication problems between physicians and researchers.

### Limitations

The studies included in the narrative review have limitations that should be acknowledged. Most studies are interview or questionnaire studies using a retrospective design. In this design there can be uncertainty whether the parents' and physicians' recollections were accurate representations of how they felt and what their thoughts were at the time of diagnosis and inclusion in a trial. Only 3 studies used a prospective design (see Table 2). Most studies have small sample sizes. Future research with larger samples and a prospective design will be able to ascertain the relationship between the specifics of the informed consent discussion and parental and physician recollection.

## Conclusion

There is an urgent need for high-quality research in children, to ensure that drugs used in the pediatric setting are both safe and effective [63]. Pediatricians must often rely on evidence that has been generated in adult populations [64], although both the safety and efficacy profiles of drugs may be significantly different for children [65]. Therefore it is of vital importance to enroll children with cancer in clinical studies. The pediatric oncology practice shows that general implementation of clinical research continuously improves outcomes for children with cancer. However, when research and clinical care coincide as much as in the pediatric oncology setting, several ethical problems can come up.

Firstly, parental equipoise is almost never reached. Parents cannot (and are not supposed to) think beyond the scope of the best interests of their child. To consider the goals of research *per se *is very difficult, if not impossible for them. Due to the diminishing boundary between research and care, parents are confronted with alleged options and treatment choices, which eventually turn out to be only accessible through research. Some parents are confronted with anticipated remorse when not participating in (promising) research. Others fear trials because of the experimental nature. Many experience a lack of freedom to reject participation. Secondly, the therapeutic misconception may endanger a correct assessment of the pros and cons of participation, because parents might inaccurately attribute therapeutic intent to research procedures. Their focus on the therapeutic effect may hamper understanding of the research purposes. All this could lead to a feeling of emotional stress and limited voluntariness that is reinforced by the trust that is often inherent to the relationship between physician and parents, especially in pediatric oncology [66].

Physicians too are constrained in their options because of their conviction that research constitutes the best available treatment, thereby passing over the greater uncertainty of the risk-benefit ratio as compared to standard medical care.

The challenges that a lack of parental equipoise and the therapeutic misconception pose may be very difficult to overcome. Thorough attention to the quality of communication of research information could improve understanding of the research perspective [16,67]. In Table 3 we summarize points of awareness with respect to research discussions and give recommendations to improve communication. But even in the case of an enormous communication effort, the question remains whether it is truly possible to explain the nature of research and thereby overcome the emotional conflict of parents who feel responsible for their child's wellbeing. Future research should focus on special efforts that might achieve this.

**Table 3:** Awareness points and recommendations for communication in relation to type of research

Type of research	Awareness*	Recommendations for communication
**Randomized Controlled Trials (RCT)**	Confidentiality and privacy The potential for a therapeutic misconception: - differences between clinical research and standard care - potential conflict between research and treatment goals Consent to treatment does not imply consent to an RCT	Provide information about collection of data and measures taken to protect confidentiality and privacy. Clarify how the physician-investigator/patient-subject relationship differs from the traditional physician-patient relationship. Mention alternatives to research participation explicitly. Discuss clinical and parental equipoise. Mention voluntariness. Indicate research interventions that are solely performed to measure trial outcomes. Assure freedom to withdraw from the study. Discuss research participation with subjects *before, during and after *the study. Ask explicit consent with a separate consent form than the consent form for treatment. Ask consent for treatment first, and for research later, preferably by a different person than the treating physician, for example a research nurse (with the opportunity to consult with the treating physician)

**Clinical Controlled Trial (CCT)**	Confidentiality and privacy Consent to treatment does not imply consent to collect patient data	Provide information about collection of data and measures taken to protect confidentiality and privacy. Ask explicit consent with a separate consent form than the consent form for treatment. Ask consent for treatment first, and for research later, preferably by a different person than the treating physician, for example a research nurse (with the opportunity to consult with the treating physician)

**Laboratory research using tissue from patients**	Confidentiality and privacy No therapeutic goal; completely distinct from therapeutic interventions The obligation of non-maleficence in this setting differs from that in clinical medicine Consent to treatment does not imply consent to using or storing human tissues for research purposes	Provide information about collection of data and measures taken to protect confidentiality and privacy. Mention voluntariness. Indicate that all research interventions to gain tissues are solely performed to gain scientific knowledge. Indicate that risks to a patient are justified not because they are outweighed by potential benefits to the patient, but because they are outweighed by the value of the knowledge to be gained from the research. Ask explicit consent with a separate consent form than the consent form for treatment. Ask consent for treatment first, and for research later, preferably by a different person than the treating physician, for example a research nurse (with the opportunity to consult with the treating physician)

* Confidentiality and privacy are not explicitly discussed in this paper. Apparently, parents and physicians do not experience these concepts as problematic or are not aware of them. Still, confidentiality and privacy are basic concepts in research ethics and should be discussed with parents. For completeness they are mentioned in the Table, in order for the Table to be used as a list to 'tick off' when communicating research with parents.

As it stands today, physicians are bound to react to the individual needs and expectations of parents and children within the context of research. The physician-investigator needs to be convinced that the best interests of the child are warranted. This means that both a therapeutic *and *a scientific orientation are appropriate, and the physician needs to shuttle between clinical care duties and research duties. To do this ethically, he continuously needs to be aware of the potential conflict between research and treatment goals (see Table 3). Having considered all this, professional integrity requires the physician to treat both the patient's interests and the scientific interests in an honest way without backsliding into a form of therapeutic misconception. After all, he has committed himself to serve both.

## List of Abbreviations

ALL: Acute Lymphoblastic Leukemia; CCT: Controlled Clinical Trial; DSMC: Data and Safety Monitoring Committee; RCT: Randomized Controlled Trial

## Competing interests

The authors declare that they have no competing interests.

## Authors' contributions

All authors read and approved the final manuscript.

MCV carried out the review and completed the manuscript. MH, DPE and EL participated in the review and helped to complete final manuscript. JMW, DB and GJKL gave valuable insights in pediatric oncology practice and helped to complete the final manuscript.

## Pre-publication history

The pre-publication history for this paper can be accessed here:

http://www.biomedcentral.com/1472-6939/12/18/prepub
